# Childhood maltreatment is linked to larger preferred interpersonal distances towards friends and strangers across the globe

**DOI:** 10.1038/s41398-024-02980-2

**Published:** 2024-08-23

**Authors:** Shilat Haim-Nachum, Marie R. Sopp, Antonia M. Lüönd, Nimrah Afzal, Fredrik Åhs, Antje-Kathrin Allgaier, Adrián Arévalo, Christian Asongwe, Rahel Bachem, Stefanie R. Balle, Habte Belete, Tilahun Belete Mossie, Azi Berzengi, Necip Capraz, Deniz Ceylan, Daniel Dukes, Aziz Essadek, Natalia E. Fares-Otero, Sarah L. Halligan, Alla Hemi, Naved Iqbal, Laura Jobson, Einat Levy-Gigi, Chantal Martin-Soelch, Tanja Michael, Misari Oe, Miranda Olff, Helena Örnkloo, Krithika Prakash, Sarah M. Quaatz, Vijaya Raghavan, Muniarajan Ramakrishnan, Dorota Reis, Vedat Şar, Ulrich Schnyder, Soraya Seedat, Ibtihal Najm Shihab, Susilkumar Vandhana, Dany Laure Wadji, Rachel Wamser, Reut Zabag, Georgina Spies, Monique C. Pfaltz

**Affiliations:** 1 Faculty of Education, Bar-Ilan University, Ramat-Gan, Israel; 2 Department of Psychiatry, Columbia University, New York, USA; 3 Division of Clinical Psychology and Psychotherapy, Saarland University, Saarbrücken, Germany; 4 Department of Consultation-Liaison Psychiatry and Psychosomatic Medicine, University Hospital, University of Zurich, Zurich, Switzerland; 5 Department of Adult Psychiatry and Psychotherapy, University Hospital of Psychiatry Zurich, Zurich, Switzerland; 6 Department of Psychology, University of Bath, Bath, UK; 7 Department of Psychology and Social Work, Mid Sweden University, Östersund, Sweden; 8 Department of Psychology, University of the Bundeswehr Munich, Munich, Germany; 9 Universidad de Piura, Facultad de Medicina, Lima, Peru; 10 Universidad Nacional Mayor de San Marcos, Facultad de Medicina “San Fernando”, Lima, Peru; 11 Department of History, Faculty of Arts, University of Bamenda, Bamenda, Cameroon; 12 Department of Psychology, University of Zurich, Zurich, Switzerland; 13 Department of Psychiatry, College of Medicine and Health Sciences, Bahir Dar University, Bahir Dar, Ethiopia; 14 Department of Clinical Psychology and Psychological Therapies, University of East Anglia, Norwich, UK; 15 Private Practice, Istanbul, Turkey; 16 Department of Psychiatry, Koç University School of Medicine, Istanbul, Turkey; 17 Swiss Center for Affective Sciences, University of Geneva, Geneva, Switzerland; 18 Interpsy EA4432, University of Lorraine, Nancy, France; 19 Hôpitaux de Saint-Maurice, Saint-Maurice, France; 20 Bipolar and Depressive Disorders Unit, Department of Psychiatry and Psychology, Hospital Clínic, Institute of Neurosciences (UBNeuro), Department of Medicine, Faculty of Medicine and Health Sciences, University of Barcelona (UB), Barcelona, Catalonia Spain; 21 Fundació Clínic per a la Recerca Biomèdica (FCRB), Institut d’Investigacions Biomèdiques August Pi i Sunyer (IDIBAPS), Network Centre for Biomedical Research in Mental Health (CIBERSAM), Health Institute Carlos III (ISCIII), Barcelona, Catalonia Spain; 22 Department of Psychiatry and Mental Health, University of Cape Town, Cape Town, South Africa; 23 Department of Psychiatry, Faculty of Medicine and Health Sciences, Stellenbosch University, Stellenbosch, South Africa; 24 Department of Psychology, Jamia Millia Islamia, New Delhi, India; 25 School of Psychological Sciences, Turner Institute for Brain and Mental Health, Monash University, Clayton, Australia; 26 The Gonda Multidisciplinary Brain Research Center, Bar-Ilan University, Ramat-Gan, Israel; 27 Department of Psychology, University of Fribourg, Fribourg, Switzerland; 28 Department of Neuropsychiatry, School of Medicine, Kurume University, Kurume, Japan; 29 Department of Psychiatry, Amsterdam Neuroscience & Public Health, Amsterdam UMC, University of Amsterdam, Amsterdam, The Netherlands; 30 ARQ National Psychotrauma Centre, Diemen, The Netherlands; 31 Department of Psychology, Eastern Michigan University, Ypsilanti, USA; 32 Schizophrenia Research Foundation, Chennai, Tamil Nadu India; 33 Research Group Applied Statistical Modeling, Department of Psychology, Saarland University, Saarbrücken, Germany; 34 South African Research PTSD Programme of Excellence, Department of Psychiatry, Faculty of Medicine and Health Sciences, Stellenbosch University, Cape Town, South Africa; 35 South African Medical Research Council / Stellenbosch University Genomics of Brain Disorders Research Unit, Department of Psychiatry, Stellenbosch University, Cape Town, South Africa; 36 Al-Kitab University, Kirkuk, Iraq; 37 Kanchi Kamakoti CHILDS Trust Hospital (KKCTH), Nungambakkam, Chennai, Tamil Nadu India; 38 Saveetha Medical College and Hospital, Saveetha Institute of Medical & Technical Sciences (SIMATS), Thandalam, Chennai, Tamil Nadu India; 39 Department of Educational and Counselling Psychology, McGill University, Montreal, Canada; 40 Psychological Sciences Faculty, University of Missouri – St. Louis, St. Louis, MO USA; 41 Department of Psychology, Bar-Ilan University, Ramat-Gan, Israel; 42 Department of Psychology, Yale University, New Haven, CT USA

**Keywords:** Pathogenesis, Human behaviour

## Abstract

Childhood maltreatment (CM) is thought to be associated with altered responses to social stimuli and interpersonal signals. However, limited evidence exists that CM is linked to larger comfortable interpersonal distance (CID) – the physical distance humans prefer towards others during social interactions. However, no previous study has investigated this association in a comprehensive sample, yielding sufficient statistical power. Moreover, preliminary findings are limited to the European region. Finally, it is unclear how CM affects CID towards different interaction partners, and whether CID is linked to social functioning and attachment. To address these outstanding issues, adults (*N* = 2986) from diverse cultures and socio-economic strata completed a reaction time task measuring CID towards an approaching stranger and friend. Higher CM was linked to a larger CID towards both friends and strangers. Moreover, insecure attachment and less social support were associated with larger CID. These findings demonstrate for the first time that CM affects CID across countries and cultures, highlighting the robustness of this association.

## Introduction

Childhood maltreatment (CM, i.e., abuse or neglect of children and adolescents by their caregivers) is a global problem. Depending on gender and continent, prevalence rates vary between 6-61% (emotional abuse), 22–60% (physical abuse), 6–27% (sexual abuse), and 17–65% (neglect) [1]. Individuals exposed to CM are at increased risk for mental and physical disorders [2]. Moreover, CM has been linked to social dysfunction, including isolation, withdrawal [3], and an increased risk of affected children to be bullied, victimized [4], and rejected by peers [5]. CM has further been linked to low levels of social support [6], elevated rates of separation and divorce, problems related to parenting, intimate partner aggression perpetration and victimization [7–9], loneliness, and social isolation [10].

Research assessing factors that might underlie problems in social functioning points to altered responses to different types of social stimuli and situations. For example, compared to unexposed adults, adults exposed to CM seem to detect facial expressions of happiness less easily but recognize negative facial expressions more easily, rapidly, and at lower intensity [11, 12]. Moreover, they tend to interpret neutral facial expressions as negative [13–15] and were found to respond with discomfort and increased neural reactivity to social touch [16]. Similarly, enhanced amygdala responses to negative facial expressions [e.g., 17] and a link between CM and neural hyperreactivity to unfamiliar neutral faces were found [18]. Based on adverse interpersonal learning experiences and given a generally heightened threat sensitivity [19, 20], individuals exposed to CM might thus respond negatively to a broad range of social stimuli. In line with this assumption, comfortable interpersonal distance is an important factor that appears to be affected by CM.

The physical distance humans prefer towards others which cannot be intruded without causing emotional and physical discomfort [21, 22] is referred to as interpersonal distance [23]. The distance that a specific person feels comfortable with (comfortable interpersonal distance, CID) is typically assessed by asking participants to stop an approaching person when they start to feel uncomfortable (stop-distance paradigm) [22]. The development of CID takes place during childhood [24] and varies, depending on the relationship to the approaching person, age and gender of the approached person [25], culture [e.g., 26, 27], and temperature within a geographical region [25]. For example, with increasing living density (more individuals per room), there seem to be differences in the perception of one’s home as crowded [26], which have been linked [27] to contact vs. noncontact [26] and to collectivistic vs. individualistic cultures [28]. Furthermore, individuals from East-Asian regions that are considered non-contact cultures have been found to prefer larger distances in a virtual task compared to individuals from European regions that are considered contact cultures [29]. Similar results have also been reported by Beaulieu [26] and Sicorello and colleagues [30]. While these differences in interpersonal distance preferences are quite well documented, reasons as to why they arise are largely unclear and further research is needed.

Consistent with the assumption that maintaining a CID serves to protect against dangers to one’s physical and emotional well-being [31], larger CID has further been linked to person-related factors such as higher levels of trait anxiety [32]. Relatedly, Cole et al. [33] showed that adults perceive threatening stimuli, including other persons, to be closer than non-threatening stimuli.

Research on the association between CM and CID is scarce. Using the stop-distance procedure, studies found an increased CID in physically abused children [34] and adults with mixed types of CM [16, 35]. This finding was replicated by Lüönd et al. [35] and by Hautle et al. [36], with mixed findings regarding the impact of depressive symptoms: Lüönd et al. [35] found that in adults with CM and symptoms of depression, all subtypes of CM were linked to a larger CID. In the absence of depressive symptoms, only adults exposed to emotional abuse showed an increased CID. In contrast, Hautle et al. [36] found that all subtypes of CM were related to a larger CID, independent of symptoms of depression.

Overall, previous research thus suggests that CM is related to larger CID and discomfort with physical proximity to others, with distinct findings for different subtypes of CM. However, sample sizes of existing studies are small, limiting reliability and conclusions that can be drawn. Moreover, existing findings stem from Croatia [34], Germany [16], and Switzerland [35, 36], i.e., from limited geographical and cultural contexts, limiting generalizability. The complete absence of transcultural research and the lack of studies involving populations outside of Europe is problematic, given cultural and geographical effects on the processing of socio-emotional signals in general [e.g., 37, 38] and on CID in particular [25–27, 39]. Finally, unlike other research on CID [e.g., 40], all of the previous studies on CID in CM have focused exclusively on CID towards strangers [16, 34–36]. It is thus unknown whether larger CID in individuals exposed to CM is restricted to strangers or extends to known others, as was found for individuals with high levels of social anxiety [41] and for individuals suffering from adjustment disorder with depressed mood [42].

The first aim of this study was to replicate the association between CM levels and CID in a large multinational sample. We hypothesized that higher CM levels would predict larger CID toward strangers (Hypothesis 1). Second, we aimed to investigate whether this relationship extended to CID towards friends (Hypothesis 2). Third, we explored potential differences in the associations between CM and CID for friends and strangers by examining the interaction between CM and the approaching individual (stranger, friend; Hypothesis 3). Beyond these hypotheses, we examined whether the association between CM and CID could be generalized across countries. We further hypothesized that the associations mentioned in Hypotheses 1 and 2 are present for all subtypes of CM (Hypothesis 4). Known correlates of CM and CID (i.e., gender, social anxiety, depressive symptoms, symptoms of posttraumatic stress disorder (PTSD), and COVID-19-related anxiety) were introduced as covariates in all of these analyses.

Finally, given the broad impairments in social functioning in individuals affected by CM, we explored whether CID is related to different aspects of social functioning in real life. Insecure attachment in particular is known to be linked to CM [43] and to alterations in CID [23, 44, 45]. Thus, we hypothesized that lower levels of social support, higher levels of social strain, and insecure (anxious and avoidant) attachment would each individually predict larger CID toward friends and strangers (Hypothesis 5).

## Results

### Sample characteristics

A total of 67.31% of the sample experienced any type of CM (Emotional abuse: 39.8%, Physical abuse: 20%, Sexual abuse: 29.3%, Physical neglect: 37.3%, Emotional neglect: 39.7%). 89.8% of the sample experienced at least one potentially traumatic event. 7.04% met the cut-off for PTSD only and 9.76% met the cut-off for both PTSD and C-PTSD. 50.5% reported mild-to-moderate depression and 12.4% reported (moderately) severe depression. 35.2% met the cut-off for social anxiety disorder and generalized social anxiety. Descriptive statistics of the CID task (per country) are reported in Supplementary Table 1.

#### Hypotheses 1, 2, & 3: Link between CM total score and CID

**Fig. 1:**
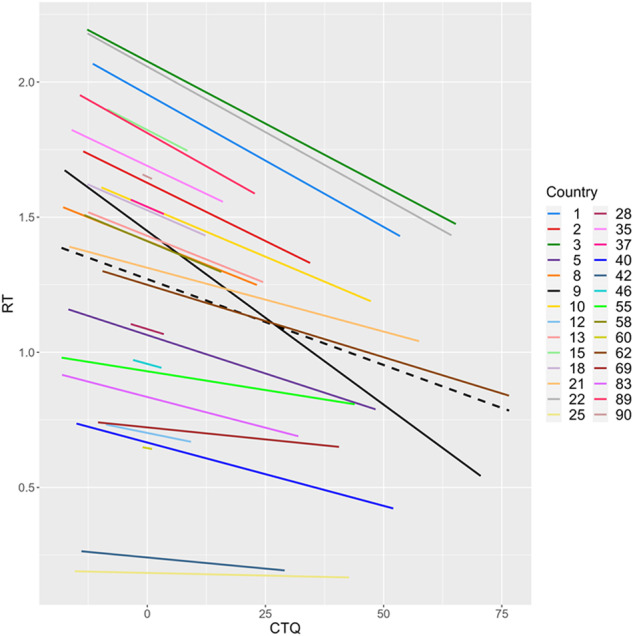
Association between childhood maltreatment (CM) and comfortable interpersonal distance (CID) in different countries. Individual regression lines of countries are weighted according to the general trend in the full sample. Regression lines are only shown for countries with *n* > 1, CTQ Childhood Trauma Questionnaire.

Observations were non-independent as reflected in an ICC of 0.88. The model including the covariates fitted our data significantly better than the baseline model (χ^*2*^diff(5) = 17.01, *p* = 0.005). Moreover, including a random slope for the approaching individual further improved model fit (χ^*2*^diff (2) = 25114.50, *p* < 0.001). However, including a random slope for CM resulted in no further improvements of model fit (χ^*2*^diff(2) = 5.46, *p* = 0.065). Thus, the effect of the approaching individual on CID varied substantially between participants, whereas this was not the case for the effect of CM scores on CID (see Fig. 1). Table 1 provides an overview of intercepts and slopes as well as estimated variance of fixed effects accounted for by each model.

**Table 1:** Model summaries of linear mixed model analyses for Hypotheses 1, 2, & 3.

	Model 1 Random Intercept			Model 2 + Covariates			Model 3 + Random Slope AI			Model 4 + Random Slope CTQ		
*Predictors*	*Estimates*	*CI*	*p*	*Estimates*	*CI*	*p*	*Estimates*	*CI*	*p*	*Estimates*	*CI*	*p*
(Intercept)	1.28	1.05–1.51	<0.001	1.28	1.05–1.51	<0.001	1.28	1.06–1.51	<0.001	1.29	1.06–1.51	<0.001
CTQ	−0.01	−0.01 to −0.01	<0.001	−0.01	−0.01 to −0.00	<0.001	−0.01	−0.01 to −0.00	<0.001	−0.01	−0.01 to −0.00	0.002
AI	0.54	0.53–0.54	<0.001	0.54	0.53–0.54	<0.001	0.53	0.52–0.55	<0.001	0.53	0.52 to 0.55	<0.001
Country-level CTQ	0.01	−0.02 to 0.03	0.675	0.01	−0.02 to 0.03	0.673	0.00	−0.02 to 0.03	0.720	0.00	−0.02 to 0.03	0.781
CTQ:approaching individual	0.00	0.00 – 0.00	0.003	0.00	0.00 – 0.00	0.003	0.00	−0.00 to 0.00	0.419	0.00	−0.00 to 0.00	0.419
FCQ				0.00	−0.00 – 0.01	0.527	0.00	−0.00 to 0.01	0.466	0.00	−0.00 to 0.01	0.447
Gender				0.07	−0.02 to 0.15	0.115	0.06	−0.02 to 0.14	0.143	0.06	−0.02 to 0.15	0.124
PHQ-9				−0.01	−0.01 to 0.00	0.157	−0.01	−0.01 to 0.00	0.150	−0.01	−0.01 to 0.00	0.149
MINI-SPIN				0.02	0.01 to 0.04	<0.001	0.02	0.01 to 0.04	<0.001	0.02	0.01 to 0.04	<0.001
ITQ – PTSD				−0.00	−0.01 to 0.00	0.438	−0.00	−0.01 to 0.00	0.423	−0.00	−0.01 to 0.00	0.393
**Random effects**												
σ^2^	0.25			0.25			0.20			0.20		
τ_00_	0.99 _Country:ID_			0.98 _Country:ID_			0.99 _Country:ID_			0.98 _Country:ID_		
	0.35 _Country_			0.35 _Country_			0.34 _Country_			0.34 _Country_		
**Random effects**												
τ_11_							0.22 _Country:ID.AI_			0.22 _Country:ID.AI_		
										0.00 _Country.CTQ_		
ρ_01_							0.05 _Country:ID_			0.06 _Country:ID_		
										−0.59 _Country_		
ICC	0.84			0.84			0.88			0.88		
Marginal *R*^*2*^	0.050			0.053			0.053			0.050		

*CTQ* Childhood Trauma Questionnaire, *FCQ* Fear of the Coronavirus Questionnaire, *PHQ-9* Patient Health Questionnaire – 9, *MINI-SPIN* Mini-Social Phobia Inventory, *ITQ* International Trauma Questionnaire, *PTSD* Posttraumatic Stress Disorder, *ID* Participant ID, *ICC* Intraclass correlation.

In the final model (Model 3), fixed effects were estimated to account for 5.3% of variance in CID. As expected, the model yielded a significant effect of the approaching individual on CID, reflecting that participants preferred larger distances towards strangers than towards friends (β = 0.21, *p* < 0.001). Critically, a significant effect of the CM score emerged, indicating that individuals with higher levels of CM showed larger CID than individuals with lower levels of CM (β = −0.08, *p* < 0.001). The CM x the approaching individual interaction failed to reach significance (β = 0.003, *p* = 0.403). Accordingly, simple slopes analyses revealed significant and similarly sized associations between CM scores and CID to friends (*B* = −0.0082, *p* < 0.001) and strangers (*B* = −0.0077, *p* < 0.001). In contrast to our prediction, a significant effect of the social anxiety score was evident, reflecting that participants with higher levels of social anxiety preferred shorter distances towards others than those with lower levels of social anxiety (β = 0.06, *p* < 0.001).

#### Hypothesis 4: Associations between CM subscale scores and CID

Separate analyses for each subscale revealed significant effects of physical abuse (β = −0.09, *p* < 0.001), sexual abuse (β = −0.04, *p* = 0.019), emotional neglect (β = −0.04, *p* = 0.031), and physical neglect (β = −0.12, *p* < 0.001) on CID. All effects reflected that higher levels of abuse/neglect were linked with larger CID. Moreover, effects were most pronounced for the physical domain (i.e., physical abuse and physical neglect). No significant effect emerged for emotional abuse (β = −0.03, *p* < 0.110). Detailed model comparisons and statistics are provided in the Supplementary File.

#### Hypotheses 5: Associations between socio-emotional functioning and CID

Analyses revealed significant effects of social support by significant others (β = 0.22, *p* < 0.001), anxious attachment (β = −0.12, *p* < 0.001), and avoidant attachment (β = −0.09, *p* < 0.001) on CID, whereby less social support, more anxious, and more avoidant attachment were linked to greater CID. Significant support by family (β = −0.04, *p* = 0.058) and friends (β = −0.02, *p* = 0.243) and social strain (β = −0.03, *p* = 0.066) had no significant impact on CID. The fixed effects were estimated to account for 2.3% of variance in CID. Table 2 provides an overview of intercepts and slopes.

**Table 2:** Model summary of linear mixed model analyses for Hypothesis 5.

Random intercept model			
Predictors	Estimates	*CI*	*p*
(Intercept)	1.29	1.06–1.52	<0.001
MSPSS – SO	0.21	0.16−0.25	<0.001
MSPSS – FR	0.02	−0.01 to 0.05	0.245
MSPSS – FA	−0.03	−0.06 to 0.00	0.059
ECR-S – ANX	−0.02	−0.03 to −0.01	<0.001
**Random intercept model**			
Predictors	Estimates	*CI*	*p*
ECR-S – AVO	−0.01	−0.02 to −0.01	<0.001
BSRS	−0.01	−0.02 to 0.00	0.065
**Random Effects**			
σ^2^	0.98		
τ_00 Country_	0.34		
ICC	0.26		
Marginal *R*^*2*^	0.023		

*MSPSS* Multidimensional Scale of Perceived Social Support, *SO* Significant Others, *FR* Friends, *FA* Family, *ECR-S* Experiences in Close Relationship Scale – Short Form, *Anx* Anxiety, *Avo* Avoidance, *BSRS* Bergen Social Relationships Scale, *ICC* Intraclass correlation.

## Discussion

This study investigated the relationship between CM and CID towards friends and strangers in 2986 adults with varying cultural backgrounds, residing in various countries. Overall, higher levels of different types of CM were linked to larger CID towards both friends and strangers. Analyses further suggested that this effect was comparable across countries (i.e., lack of model improvement by including CM random slope). Distal outcomes analyses showed that insecure attachment styles were associated with a larger CID. Furthermore, while levels of interpersonal stress were unrelated to CID, individuals reporting less social support preferred a larger CID. No significant gender differences were found in any of the analyses.

In line with our first hypothesis, CM predicted larger CID towards strangers. Although effect sizes were small, we were able to demonstrate significant associations between CM and larger CID towards strangers across countries. Thus, while other long-term effects of CM such as depressive symptoms and borderline personality disorder seem to be affected by cultural background [46–48], the effect of CM on CID might more strongly and universally be driven by factors such as CM induced changes in anatomical structures associated with CID such as the amygdala [19, 20, 49]. Future research should investigate whether CID is associated with behavioral and social problems related to CM such as loneliness or social isolation [10] and whether the impact of CID differs depending on culture.

Furthermore, our study shows for the first time that associations between CM and CID are also present when an imagined friend was approaching. Given the association of CM with parenting stress and marital separation [7, 9, 50], larger CID towards close others might contribute to disruptions in relationships experienced by adult survivors of CM, e.g., through discomfort associated with social touch [16]. Intact social relationships, however, have the potential to protect victims of CM from developing both physical and mental disorders [51, 52] and to promote well-being in a broad sense [2]. Individuals exposed to CM might thus benefit from interventions aimed at improving social functioning. Importantly, associations between CM and CID did not differ between the friends and stranger condition, even though the approaching figure’s effect on CID varied substantially between countries. This highlights that in the case of CM, CID might be generally, i.e., across different interaction partners, elevated, potentially impairing the development and maintenance of supportive social relationships [2, 3, 7].

In line with this assumption, we found that individuals reporting less social support by significant others preferred larger CID. However, interpersonal stress was not associated with CID. One factor that might explain this difference is loneliness, which has been associated with CM [3], lower relationship satisfaction [53], and larger CID [54, 55]. Notably, Saporta et al. [55] found that while chronic loneliness is linked to increased CID, situational loneliness (e.g., during the COVID-19 pandemic) is linked to decreased CID. Thus, situational loneliness might serve an adaptive function towards social connectedness [56], whereas individuals who are chronically lonely tend to avoid social contact [57]. Although our measures of social support and interpersonal stress did not ask for the duration of the reported experiences [58, 59], social support and social strain might reflect levels of chronic and acute loneliness to a different degree. The fact that no effects of COVID-19 or fear thereof were found in our study might be mainly due to the low reliability of our corresponding measures. To better understand the relationship between CM, CID, and both social support and interpersonal stress, future studies should thus collect information on the chronicity of these experiences and on loneliness.

Individuals affected by CM tend to develop insecure (anxious or avoidant) attachment styles [60, 61]. Given the protective role of CID, we had hypothesized that participants high in attachment avoidance and anxiety would prefer larger CID, which was confirmed by our results. This is in line with previous findings of larger CID in adults with insecure attachment styles [23, 44, 45] and might suggest that individuals with CM show a lower threshold to respond with unpleasant feelings and corresponding physiological states [21, 22] to the presence of others. These responses might affect their behavior, including non-verbal signals (e.g., body posture), and negatively impact social interactions. Interventions might thus aim at increasing a sense of safety and security, e.g. through body-oriented approaches [62].

Contrasting previous findings [36, 41, 63, 64], we found that individuals with higher levels of social anxiety preferred smaller CID compared to those with lower levels of social anxiety. One reason for this surprising and counterintuitive finding may be related to the CID task itself, given that it was performed in a seemingly neutral virtual environment with no additional social clues that may be feared by those with pronounced social anxiety (e.g. eye gaze, emotional expression). Gilbert [65] suggested that individuals would show more affiliative behavior when they feel socially safe, which is in line with research documenting reduced security-seeking and increased affiliative behavior in individuals with social anxiety when situational felt security was high [66]. Therefore, when conducting the virtual CID task, participants with higher levels of social anxiety possibly felt safe enough to approach the interaction partner. Furthermore, by maintaining closer proximity to others, they may feel better able to monitor and manage social interactions, reducing feelings of uncertainty or vulnerability. In this population, choosing a smaller distance may thus have evoked a sense of control over their environment. However, it should also be pointed out that we assessed social anxiety using the Mini-SPIN, a three-item measure for social anxiety disorder [67], which is limited due to its brevity. Given that previous studies were either conducted in clinical populations [63] or used a more extensive questionnaire to assess social anxiety [36, 41, 64], these results should be replicated, e.g., in clinical populations, and interpreted with caution.

Regarding the impact of CM subtypes on CID, our results are partly in line with Hautle et al. [36] who found associations of CM with all subtypes of CM, and suggest that both abuse and neglect have the potential to interfere with socioemotional development [68–70]. Yet, in contrast to Lüönd et al. [35], we found that emotional abuse was not associated with larger CID. Although subtypes frequently co-occur [70], specific neural and behavioral differences between abuse and neglect have been documented [20, 71]. As the experience of neglect is distinct from abuse [72] and behavioral differences between abused and neglected adults are documented [73], individuals who predominantly experienced neglect might also show different nonverbal reactions to intrusions of their personal space compared to those who predominantly experienced abuse. For example, they might feel insecure when confronted with (too much) physical closeness as it is something they are not familiar with. Conversely, abused individuals might associate physical closeness with potential danger of being hurt and display protective, aggressive non-verbal behavior [74]. Future studies should thus investigate whether nonverbal behavior associated with intrusion of personal space differs depending upon the subtype of CM.

### Strengths and limitations

We included data from 43 countries, representing a wide range of cultures, including individuals from non-WEIRD^1^ countries. However, while we statistically accounted for varying sample sizes between countries, future studies should aim for more balanced sample sizes. Furthermore, using a virtual reality paradigm might help to assess and control for the impact of age and gender of the approaching figure [75], which, to simplify our paradigm, we did not attempt. Future studies may also examine habituation across trials, since this could shed further light on the alterations of interpersonal distance regulation in individuals with CM. Unfortunately, the necessary data was not available to conduct such analyses in the current study. Finally, given that we used an experimental task, results should be replicated using closer to real-life conditions [76]. Nevertheless, it is important to reiterate that the CID task is a validated [77], often-used procedure [40, 41, 63, 64] that correlates with real-life distance between participants and strangers [77, 78]. Moreover, studies using both a computerized version of the CID task and the stop-distance method did not report different results between the two [64]. Nevertheless, the task we used relies on imaginary skills. Individuals vary in these skills [79, 80] and such variations between individuals and – potentially – between countries, may have contributed to error variance and thus to the rather small effects we found. Moreover, despite evidence for the generalization of findings from the online task to real-world behavior [64, 77, 78], we cannot rule out that the nature of the task we used (absence of real persons, reliance on imaginary skills, differences in the speed of participants’ internet connections) may have affected the reliability as well as the ecological validity of our measures, possibly explaining larger effects sizes in studies with real-life encounters [e.g., 35, 36].

## Conclusion

Our findings suggest that child maltreatment (CM) is linked to distorted regulation of comfortable interpersonal distance (CID) across cultures, with a history of CM being linked to larger CID towards strangers and close others. Furthermore, CID was larger in individuals with insecure attachment and in those reporting low levels of social support. Given the importance of social relationships on mental health, exploring ways to support individuals affected by CM in the regulation of interpersonal distance might contribute to the development of effective preventive and therapeutic interventions. Such research would be especially important considering the similar effects we found across cultures.

## Methods

### Participants

As part of the Child Trauma research group (https://www.global-psychotrauma.net/cm-sec) of the Global Collaboration on Traumatic Stress [81, 82], a total of 3656 participants were recruited from the general population around the globe (for a detailed description of the countries, see Table 3). Selection of countries focused on representation of diverse cultures to maximize socio-economic variation. Sample size was based on the detection of medium-sized associations (*r* = 0.15; one sided) between CM and interpersonal distance, with a power of 0.80, using G*Power software [83]. Therefore, we initially sought to recruit a minimum of 270 participants per country. Unexpectedly, response rates differed widely across countries and several individuals who were not from the predefined target populations (Australia, Cameroon, Ethiopia, France, Germany, India, Iraq, Israel, Japan, Peru, South Africa, Spain, Sweden, Switzerland, Turkey, UK, and USA) completed the study. However, widely varying sample sizes were accounted for by means of multilevel (ML) analyses.

**Table 3:** List of countries and participants.

Country	*n*
South Africa	475
India	401
Switzerland	400
Israel	314
Germany	292
Sweden	228
UK	172
Turkey	168
France	157
Japan	88
Iraq	64
Cameroon	45
US	45
Spain	30
Ethiopia	22
Peru	21
Australia	19
Austria	4
Namibia	4
Zimbabwe	4
Canada	3
Brazil	2
Greece	2
Kenya	2
Kuwait	2
Lesotho	2
Liechtenstein	2
Netherlands	2
Albania	1
Columbia	1
Hong Kong	1
Iran	1
Italy	1
Malaysia	1
Mexico	1
Mozambique	1
Norway	1
Philippines	1
Russia	1
South Korea	1
Thailand	1
Uruguay	1
Zambia	1

Participants were recruited via personal contacts, online platforms and (social) media advertisements (e.g., Facebook ads, Qualtrics service). Eligible participants were at least 18 years old and had sufficient reading skills and understanding of the local language.

Participants were excluded if: 1) they did not complete a minimum of 75% valid trials of the CID task; 2) they did not complete a minimum of 50% valid trials of the CID task for each trial category (i.e., friend, stranger, speed; for details see *Assessment of preferred interpersonal distance*); 3) the variance in their reaction times (RTs) of control trials exceeded three interquartile ranges above the upper quartile. Criterion 1 and 2 were specified after data collection because participants showed an unexpected high number of missing responses during the CID task. In the preregistration, we initially intended to remove participants that did not complete all necessary measures.

In total, 670 participants were excluded based on these criteria, resulting in *n* = 2986 participants (69.2% female, *M*_age_ = 31.27, *SD*_age_ = 13.36; see Table 4 for detailed sample characteristics). Data was collected online, from October 2021 through March 2022, using the software Qualtrics (Provo, USA).

**Table 4:** Demographics and psychometric characteristics.

Variables	Mean (*SD*)
Age (years)	31.28 (13.35)
Female/Male/Else (*n*)*	2066/896/24
Education (years)	15.48 (4.06)
Social ladder rank	6.29 (1.61)
CID – friend	1.66 (1.17)
CID – stranger	1.13 (1.13)
Total CTQ score	47.35 (11.97)
Emotional abuse	8.9 (4.59)
Physical abuse	6.54 (3.05)
Sexual abuse	6.68 (3.81)
Emotional neglect	8.76 (3.9)
Physical neglect	7.37 (2.92)

*CID* Comfortable Interpersonal Distance, *CTQ* Childhood Trauma Questionnaire. *The values for Female/Male and households represent frequencies.

### Procedure

An umbrella ethics approval was obtained from Saarland University (Identification number: 21-07). In addition, local ethics approvals were obtained in countries requiring a separate application. All study procedures were preregistered in September 2021 (available at https://aspredicted.org/pc9rv.pdf). Study data can be accessed via the Open Science Framework (https://osf.io/nxrfu/?view_only=25248e7fe4174f8b88cfe14c57e19e9d). The study was offered in Afrikaans, Arabic, Amharic, English, French, German, Hebrew, Japanese, Spanish, Swedish, Turkish, and Xhosa. Before starting the experiment, participants received information on the study procedures and provided informed consent. They first completed a modified online version of the well-established CID task [41]. They then provided demographic information (age, gender, country of residence, birth country, and socioeconomic status). Finally, they completed questionnaires assessing CM levels (The Childhood Trauma Questionnaire; CTQ) [84], lifetime trauma exposure (Life Events Checklist; LEC) [85], PTSD symptoms (International Trauma Questionnaire; ITQ) [86], social anxiety (Mini-Social Phobia Inventory; MINI-SPIN) [67], depression (Patient Health Questionnaire-9; PHQ-9) [87], social support (Multidimensional Scale of Perceived Social Support; MSPSS) [59], interpersonal stress in close relationships (Bergen Social Relationships Scale; BSRS) [58], attachment styles (Experiences in Close Relationship Scale – Short Form; ECR-S) [88], and fear of COVID-19 (Fear of the Coronavirus Questionnaire; FCQ) [89] (for details see Supplementary File). Participating psychology students were given course credits.

### Measures

#### Assessment of preferred interpersonal distance

**Fig. 2:**
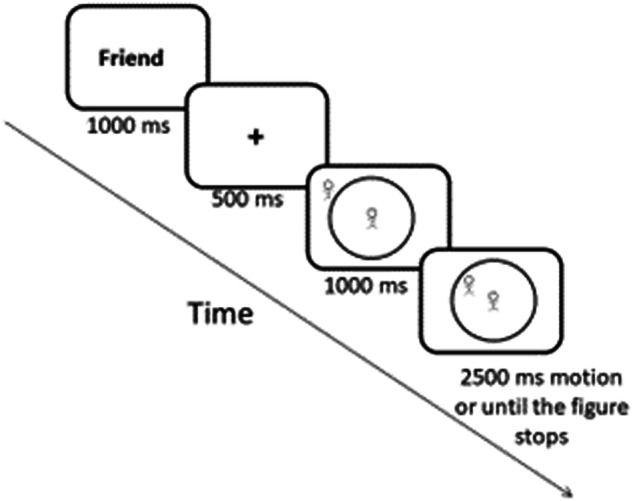
Example of an experimental trial, including instructions for participants. During the task, you will see a circular room on the screen with a line-figure standing in the center. You are instructed to imagine you are the figure. In each trial, you will see another figure at the entrance approaching you. In some of the trials, we ask you to imagine the approaching figure is a friend, and in other trials we ask you to imagine the approaching figure is a stranger. Please press the spacebar as soon as you start feeling uncomfortable with the distance between you and the approaching figure.

We programmed an online version of the CID task [41] to assess the main dependent variable, CID. This validated task has previously been used to assess both CID towards friends and strangers in different populations [e.g., 41, 90] and correlates with different interpersonal distance measures, including real-life distance between participants and strangers [77, 78]. Correlations with real-life distance measures have been reported as high as r = 0.52–0.76 [78]. Reliability is also high – as reflected in a split-half correlation of r = 0.95 and a 16-month retest-reliability of r = 0.47–70 [90]. External validity of the CID measure is further supported by its association with measures of control, comfort, need for privacy, and other personality attributes that can predict differences in inter-personal distance preferences [e.g., 23, 44, 91–93]. More recent research [64] further substantiates the generalization of the findings from the CID task to real-world distance behavior. During the task, participants see a circular room on the screen with a line-figure standing in the center. They are instructed to imagine they are the figure. In each trial, they see another figure at the entrance approaching them. In half of the trials, participants are asked to imagine the approaching figure is a friend. In the remaining trials, they are asked to imagine the approaching figure is a stranger. Thereafter, the figure representing the friend or stranger approaches the participant in the center of the room. Participants are requested to press the spacebar when they want the approaching figure to stop/as soon as they start feeling uncomfortable with the distance between themselves and the approaching figure (see example Fig. 2). The task includes eight different radii (entrances) corresponding to 0°, 45°, 90°, 135°, 180°, 225°, 270°, and 315°. Each of the two figures (stranger/friend) appears 48 times: six repetitions per radius. To account for device specific timing errors, participants completed eight intermixed control trials or “speed trials” (for a total of 56 trials) during which they saw the same room with the two figures and were instructed to press the spacebar as soon as the trial began, and the room appeared on their screen. We accounted for RTs on these trials when analysing RTs during interpersonal distance trials. Moreover, to avoid responses based on a desire to shorten the task, each trial lasted 5 s, regardless of the chosen distance.

The outcome variable was the RT, with higher RTs reflecting smaller CID. For Hypotheses 1, 2, and 3, RTs of individual trials (minus mean RTs on control trials) were subjected to the analyses. Since Hypotheses 4 and 5 did not focus on differences in RTs related to the approaching individual, RTs were collapsed across trials for the respective analyses, reducing model complexity. The mean RT (minus the mean RT on control trials) was thus subjected to analyses.

### Data analyses

A series of multilevel models was fit to investigate associations between the CTQ score, CID, and outcomes of socio-emotional functioning. The acquired data were nested in a three-level structure, such that trials (Level 1) were nested in participants (Level 2), which were nested in countries (Level 3). For Hypotheses 1, 2, and 3, ML models included the Level 2 (L2) predictor CTQ score, the Level 1 (L1) predictor approaching individual, and their interaction. In addition, we introduced country-level CTQ mean as an L3 predictor to investigate whether level of CM in a given country accounted for differences in CID between individuals. To account for further sources of variance, the Level 2 predictors Gender, MINI-SPIN score, ITQ score, PHQ score, and FCQ score were introduced as covariates. For Hypotheses 1, 2, and 3, all variables were group-mean centered on participant (L2 variables) or trial level (L1 variable – approaching individual) [94]. First, we tested a random intercept model comprising CTQ score, approaching individual, and their interaction as fixed effects (Model 1). Then, we tested the improvement of model fit after inclusion of covariates (Model 2). Thereafter, random slopes for the approaching individual (Model 3) and CTQ score (Model 4) were subsequently entered. Final model selection was based on significant improvements of model fit. Generalizability of the association between CTQ scores and CID between countries was assessed by evaluating the random slope of CTQ score. Since including this slope did not yield a significant improvement of model fit – indicating a lack of substantial between-country variability in the association between CTQ score and CID – we refrained from examining potential country-level moderators (explorative analyses mentioned in the preregistration).

Since Hypotheses 4 and 5 did not focus on effects of the approaching individual, RTs were aggregated across trials resulting in a 2-level structure with participants (L1) being nested in countries (L2). Deviating from our preregistration, we tested Hypothesis 4 by conducting separate analyses for each CTQ subscale rather than introducing these in a combined model because the high level of intercorrelation between subscales in fact precluded such analyses. For Hypothesis 4, ML models thus included the respective CTQ subscale as L1 predictor as well as Gender, MINI-SPIN score, ITQ score, PHQ score, and FCQ score as L1 covariates. In addition, we introduced country-level CTQ subscale mean as an L2 predictor to investigate whether the level of CM in a given country accounted for differences in CID between individuals. All variables were group-mean centered on participant-level [94]. In a first step, we tested a random intercept model comprising the respective CTQ subscale score (Model 1). Then, we tested the improvement of model fit after inclusion of covariates (Model 2). Thereafter, a random slope for the respective CTQ subscale (Model 3) was entered and model fit was re-evaluated. Final model selection was based on significant improvements of model fit.

For Hypothesis 5, the ML model included ECR-S subscales, MSPSS subscales, and BSRS scores as L1 predictors. All variables were group-mean centered on participant-level [94]. A random intercept model comprising all predictors was tested.

All multilevel models were fit using restricted maximum likelihood estimation and the lme4 package [95, 96] in R [97]. Interactions were probed using simple slopes techniques implemented in the R package reghelper [98]. The two-sided α level was set to 0.05 for all analyses. Degrees of freedom varied across analyses, given missing data.

### Supplementary information

Supplementary Material

## Data Availability

Study data can be accessed via the Open Science Framework (https://osf.io/nxrfu/?view_only=25248e7fe4174f8b88cfe14c57e19e9d).

## Footnotes

1 WEIRD = Western, Educated, Industrialised, Rich, and Democratic
