# Supplementary material for: Childhood maltreatment is linked to larger preferred interpersonal distances towards friends and strangers across the globe
Source: Transl Psychiatry. 2024 Aug 23;14:339. doi: 10.1038/s41398-024-02980-2 (PMC11344078; doi:10.1038/s41398-024-02980-2)
Supplement: Supplementary file 1 — Supplementary Material [file 41398_2024_2980_MOESM1_ESM.docx]

**Supplementary File**

**Description of questionnaires**

**Childhood trauma.** The *Childhood Trauma Questionnaire* (CTQ; Bernstein et al., 1994) measures the severity of five CM subscales: Emotional abuse, physical abuse, sexual abuse, emotional neglect, and physical neglect. For each item, participants respond in the context of “when you were growing up”, ranging from 1 = “never” to 5 = “very often”, producing scores of 5 to 25 for each subscale. Cut-off scores were computed according to the manual of the CTQ (Bernstein et al., 2003). Internal consistency coefficients in the current sample ranged between α = .65 (physical neglect), α = .83 (physical abuse), α = .86 (emotional neglect), α = .87 (emotional abuse), and α = .91 (sexual abuse).

**Trauma exposure.** *Life Events Checklist* (LEC; Weathers et al., 2013). Participants were screened for 16 potentially traumatic events. For each event, they indicated if it directly happened to them, they witnessed it happen to someone else; they learned about it happening to a person close to them; and/or they were exposed to it as part of their job; or if it did not apply. If more than one event occurred, participants were asked to describe the event that currently bothers them the most and estimate how long ago it happened.

**Trauma-related symptoms.** The *International Trauma Questionnaire* (ITQ; Cloitre et al., 2018) focuses on the core features of PTSD and complex PTSD (C-PTSD), screening for symptoms including re-experiencing, avoidance, and sense of current threat as well as affective dysregulation, negative self-concept, and disturbances in relationships. An additional item screens for the effect of these symptoms on participants’ functioning, including social and work-related functioning. Internal consistency in the current sample was α = .92.

**Depression.** The *Patient Health Questionnaire-9* (PHQ-9; Kroenke et al., 2001) is a brief instrument designed to identify individuals with nine depressive symptoms experienced over the past two weeks (e.g., anhedonia, sleep disturbances, low self-esteem, concentration difficulties). Responses are scored from 0 “not at all” to 3 “nearly every day”. Symptom severity was assessed by summing all item scores. Internal consistency in the current sample was α = .89.

**Social phobia.** We applied the *Mini-Social Phobia Inventory* (MINI-SPIN; Connor et al., 2001) which contains three items about avoidance of social activities and fear of embarrassment during the past week. Items are rated from 0 "not at all " to 4 "extremely"; higher scores indicate greater levels of social phobia. Internal consistency in the current sample was α = .85.

**Perceived social support.** The *Multidimensional Scale of Perceived Social Support* (MSPSS; Zimet et al., 1988) is a 12-item scale, assessing respondents’ self-perceived level of support from family, friends, and significant others. Each item is rated on a scale from 1 “very strongly disagree” to 7 “very strongly agree”. Internal consistency in the current sample ranged between α = .93 (family), α = .94 (friends), and α = .95 (significant others).

**Social stress.** The *Bergen Social Relationships Scale* (BSRS; Bancila & Mittelmark, 2009) assesses social strain (i.e., interpersonal stress that occurs in relationships with significant others who cause stress even when they do not mean to). The measure includes six types of social situations that could be seriously stressful (e.g., criticism, high demands). Response alternatives range from “does not describe me at all” to “describes me very well”, with higher scores reflecting higher levels of social stress. Internal consistency in the current sample was α = .80.

**Attachment style.** We used the *Experiences in Close Relationship Scale –* Short Form (ECR-S; Wei et al., 2007) to measure adult attachment. The scale measures how individuals generally experience close relationships (e.g., close friends, family members, romantic partners). Respondents are asked to rate their agreement with each of the 12 items on a 7 point-Likert scale. Results consist of two scores for two separate factors; attachment *anxiety* (e.g., fear of rejection, dependence on partners) and attachment *avoidance* (e.g., excessive need for self-reliance, reluctance to self-disclose). Higher scores on either or both of these factors reflect insecure attachment style. The overall internal consistency of the scale in the current study was .76 (α = .73 and α = .71, for anxiety and avoidance, respectively).

**COVID-19.** To account for effects driven by the COVID-19 pandemic, we asked participants to indicate whether they have ever tested positive for SARS-CoV-2 and which level of restrictions were in place where they lived at the time of study.

We further used the *Fear of the Coronavirus Questionnaire* (FCQ; Mertens et al., 2020) to assess the potential impact of COVID-19-related fears on interpersonal distance. Respondents rate their agreement level with eight items from 1 = “strongly disagree” to 5 = “strongly agree”. Sample items: “I am very worried about the coronavirus”, “I am taking precautions to prevent infection”, and “I am constantly following all news updates regarding the virus”. Internal consistency in the current sample was α = .51.

***Translation of questionnaires***

Questionnaires that were not available in all study languages were translated according to predefined criteria which were based on available guidelines (Wild et al., 2005). Two forward and two backward translators were used per target language. All translators were native speakers of the target language and were fluent in the source language. To generate the final translated questionnaire, both independent versions were compared with each other, and discrepancies resolved.

**Detailed Analyses of Hypothesis # 4**

***Analysis of Emotional Neglect as Predictor of CIPD***

The model including the covariates fitted our data significantly better than the baseline model (*χ^2^*diff(5) = 20.34, p = .001). Including a random slope for the CTQ Emotional Neglect (EN) subscale did not result in any further improvements of model fit (*χ^2^*diff (2) = 1.74, p = .420). Supplementary Table 1 provides an overview of intercepts and slopes as well as the estimated variance of fixed effects accounted for by each model.

In the final model (Model 2), the fixed effects were estimated to account for 0.8% of variance in CIPD. The model yielded a significant effect of the CTQ EN subscale on CIPD, indicating that individuals with higher levels of EN preferred larger distances towards others than individuals with lower levels of EN (*β* = -.04, *p* = .031). In addition, significant effects of the MINI-SPIN score (*β* = .07, *p* < .001) and the PHQ-9 score (*β* = -.03, *p* = .020) were evident, reflecting that participants with higher levels of social anxiety and lower levels of depression preferred shorter distances towards others than those with lower levels of social anxiety and higher levels of depression.

***Analysis of Physical Neglect as Predictor of CIPD***

The model including the covariates fitted our data significantly better than the baseline model (*χ^2^*diff(5) = 14.88, p = .011). Moreover, including a random slope for the CTQ Physical Neglect (PN) subscale further improved model fit (*χ^2^*diff (2) = 7.26, p = .026). Supplementary Table 2 provides an overview of intercepts and slopes as well as the estimated variance of fixed effects accounted for by each model.

In the final model (Model 3), the fixed effects were estimated to account for 2.3% of variance.

The model yielded a significant effect of the CTQ PN subscale on CIPD, indicating that individuals with higher levels of PN preferred larger distances towards others than individuals with lower levels of PN (*β* = -.12, *p* < .001). In addition, a significant effect of the MINI-SPIN score (*β* = .06, *p* < .001) emerged, reflecting that participants with higher levels of social anxiety preferred shorter distances towards others than those with lower levels of social anxiety.

***Analysis of Physical Abuse as Predictor of CIPD***

The model including the covariates fitted our data significantly better than the baseline model (*χ^2^*diff(5) = 16.27, *p* = .006). Moreover, including a random slope for the CTQ Physical Abuse (PA) subscale further improved model fit (*χ^2^*diff (2) = 9.32, p = .009). Supplementary Table 3 provides an overview of intercepts and slopes as well as the estimated variance of fixed effects accounted for by each model.

In the final model (Model 3), the fixed effects were estimated to account for 1.2% of variance. The model yielded a significant effect of the CTQ PA subscale on CIPD, indicating that individuals with higher levels of PA preferred larger distances towards others than individuals with lower levels of PA (*β* = -.09, *p* < .001). In addition, a significant effect of the MINI-SPIN score (*β* = .07, *p* < .001) emerged, reflecting that participants with higher levels of social anxiety preferred shorter distances towards others than those with lower levels of social anxiety.

***Analysis of Sexual Abuse as Predictor of CIPD***

The model including the covariates fitted our data significantly better than the baseline model (*χ^2^*diff(5) = 19.86, p = .001). Including a random slope for the CTQ Sexual Abuse (SA) subscale did not result in any further improvements of model fit (*χ^2^*diff (2) = 0.36, p = .835). Supplementary Table 4 provides an overview of intercepts and slopes as well as the estimated variance of fixed effects accounted for by each model.

In the final model (Model 2), the fixed effects were estimated to account for 0.7% of variance. The model yielded a significant effect of the CTQ SA subscale on CIPD, indicating that individuals with higher levels of SA preferred larger distances towards others than individuals with lower levels of SA (*β* = -.04, *p* = .019). In addition, significant effects of the MINI-SPIN score (*β* = .07, *p* < .001) and the PHQ-9 score (*β* = -.05, *p* = .015) were evident, reflecting that participants with higher levels of social anxiety and lower levels of depression preferred shorter distances towards others than those with lower levels of social anxiety and higher levels of depression.

***Analysis of Emotional Abuse as Predictor of CIPD***

The model including the covariates fitted our data significantly better than the baseline model (*χ^2^*diff(5) = 20.07, p = .001). Including a random slope for the CTQ Emotional Abuse (EA) subscale did not result in any further improvements of model fit (*χ^2^*diff (2) = 2.49, p = .289). Supplementary Table 5 provides an overview of intercepts and slopes as well as the estimated variance of fixed effects accounted for by each model.

In the final model (Model 2), the fixed effects were estimated to account for 1.1% of variance. The model did not yield a significant effect of the CTQ EA subscale on CIPD (*β* = -.03, *p* = .110). Significant effects of the MINI-SPIN score (*β* = .07, *p* < .001) and the PHQ-9 score (*β* = -.04, *p* = .032) were evident, reflecting that participants with higher levels of social anxiety and lower levels of depression preferred shorter distances towards others than those with lower levels of social anxiety and higher levels of depression.

| **Variables** | **South**  **Africa** | **Switzerland** | **India** | **Israel** | **Germany** | **Sweden** | **UK** | **Turkey** | **France** | **Japan** | **Iraq** |
| --- | --- | --- | --- | --- | --- | --- | --- | --- | --- | --- | --- |
| CID – friend | 1.77 (1.14) | 2.40 (0.99) | 1.22 (0.84) | 1.58 (1.1) | 2.38 (1.06) | 0.96 (1.05) | 2.37 (0.87) | 1.04 (1.08) | 1.67 (1.15( | 1.85 (0.95) | 0.38 (0.71) |
| CID – stranger | 1.19 (1.14) | 1.87 (1.00) | 0.91 (0.79) | 0.93 (1.1) | 1.84 (1.04) | 0.36 (1.01) | 1.82 (0.88) | 0.44 (0.97) | 1.09 (1.13) | 1.23 (0.92) | -.02 (0.71) |

**Supplementary Table 1.** Psychometric characteristics for CID per country, Mean (SD)

*Note.* CID = Comfortable Interpersonal Distance

**Supplementary Table 2.** Model summary of linear mixed model analyses for Hypothesis # 4 – Emotional Neglect

|  | **Model 1**  **Random Intercept** | | | **Model 2**  **+ Covariates** | | | **Model 3**  **+ Random Slope CTQ** | | |
| --- | --- | --- | --- | --- | --- | --- | --- | --- | --- |
| *Predictors* | *Estimates* | *CI* | *p* | *Estimates* | *CI* | *p* | *Estimates* | *CI* | *p* |
| (Intercept) | 1.28 | 1.05 – 1.51 | **<0.001** | 1.28 | 1.05 – 1.51 | **<0.001** | 1.28 | 1.05 – 1.51 | **<0.001** |
| CTQ_EN | -0.01 | -0.02 – -0.00 | **0.006** | -0.01 | -0.02 – -0.00 | **0.031** | -0.01 | -0.02 – 0.00 | 0.150 |
| Country-level CTQ_EN | 0.04 | -0.06 – 0.13 | 0.466 | 0.04 | -0.06 – 0.13 | 0.464 | 0.04 | -0.06 – 0.13 | 0.430 |
| FCQ |  |  |  | 0.00 | -0.00 – 0.01 | 0.498 | 0.00 | -0.00 – 0.01 | 0.521 |
| Sex |  |  |  | 0.07 | -0.01 – 0.15 | 0.103 | 0.07 | -0.01 – 0.15 | 0.092 |
| PHQ-9 |  |  |  | -0.01 | -0.02 – -0.00 | **0.020** | -0.01 | -0.02 – -0.00 | **0.023** |
| MINI-SPIN |  |  |  | 0.02 | 0.01 – 0.04 | **<0.001** | 0.02 | 0.01 – 0.04 | **<0.001** |
| ITQ – PTSD |  |  |  | -0.01 | -0.01 – 0.00 | 0.054 | -0.01 | -0.01 – 0.00 | 0.053 |
| **Random Effects** | | | | | | | | | |
| σ^2^ | 1.00 | | | 1.00 | | | 1.00 | | |
| τ_00_ | 0.35 _Country_ | | | 0.35 _Country_ | | | 0.35 _Country_ | | |
| τ_11_ |  | | |  | | | 0.00 _Country.CTQ_EN_ | | |
| ρ_01_ |  | | |  | | | -0.52 _Country_ | | |
| ICC | 0.26 | | | 0.26 | | | 0.26 | | |
| Marginal R^2^ | 0.003 | | | 0.008 | | | 0.007 | | |

# Note. CTQ = Childhood Trauma Questionnaire, EN = Emotional Neglect, PHQ = Patient Health Questionnaire, FCQ = Fear of the Coronavirus Questionnaire, PHQ-9 = Patient Health Questionnaire – 9, MINI-SPIN = Mini-Social Phobia Inventory, ITQ = International Trauma Questionnaire, ICC = Intraclass correlation.

**Supplementary Table 3.** Model summary of linear mixed model analyses for Hypothesis # 4 – Physical Neglect

|  | **Model 1**  **Random Intercept** | | | **Model 2**  **+ Covariates** | | | **Model 3**  **+ Random Slope CTQ** | | |
| --- | --- | --- | --- | --- | --- | --- | --- | --- | --- |
| *Predictors* | *Estimates* | *CI* | *p* | *Estimates* | *CI* | *p* | *Estimates* | *CI* | *p* |
| (Intercept) | 1.31 | 1.09 – 1.53 | **<0.001** | 1.31 | 1.09 – 1.53 | **<0.001** | 1.31 | 1.08 – 1.53 | **<0.001** |
| CTQ_PN | -0.05 | -0.07 – -0.04 | **<0.001** | -0.05 | -0.07 – -0.04 | **<0.001** | -0.05 | -0.07 – -0.03 | **<0.001** |
| Country-level CTQ_PN | -0.05 | -0.14 – 0.04 | 0.276 | -0.05 | -0.14 – 0.04 | 0.277 | -0.04 | -0.13 – 0.05 | 0.430 |
| FCQ |  |  |  | 0.00 | -0.00 – 0.01 | 0.481 | 0.00 | -0.00 – 0.01 | 0.485 |
| Sex |  |  |  | 0.05 | -0.03 – 0.14 | 0.199 | 0.06 | -0.03 – 0.14 | 0.180 |
| PHQ-9 |  |  |  | -0.01 | -0.01 – 0.00 | 0.091 | -0.01 | -0.01 – 0.00 | 0.101 |
| MINI-SPIN |  |  |  | 0.02 | 0.01 – 0.04 | **0.001** | 0.02 | 0.01 – 0.04 | **0.001** |
| ITQ – PTSD |  |  |  | -0.00 | -0.01 – 0.00 | 0.277 | -0.00 | -0.01 – 0.00 | 0.362 |
| **Random Effects** | | | | | | | | | |
| σ^2^ | 0.98 | | | 0.98 | | | 0.98 | | |
| τ_00_ | 0.32 _Country_ | | | 0.32 _Country_ | | | 0.33 _Country_ | | |
| τ_11_ |  | | |  | | | 0.00 _Country.CTQ_PN_ | | |
| ρ_01_ |  | | |  | | | -0.65 _Country_ | | |
| ICC | 0.24 | | | 0.24 | | | 0.25 | | |
| Marginal R^2^ | 0.019 | | | 0.023 | | | 0.019 | | |

# Note. CTQ = Childhood Trauma Questionnaire, PN = Physical Neglect, PHQ = Patient Health Questionnaire, FCQ = Fear of the Coronavirus Questionnaire, PHQ-9 = Patient Health Questionnaire – 9, MINI-SPIN = Mini-Social Phobia Inventory, ITQ = International Trauma Questionnaire, ICC = Intraclass correlation

**Supplementary Table 4.** Model summary of linear mixed model analyses for Hypothesis # 4 – Physical Abuse

|  | **Model 1**  **Random Intercept** | | | **Model 2**  **+ Covariates** | | | **Model 3**  **+ Random Slope CTQ** | | |
| --- | --- | --- | --- | --- | --- | --- | --- | --- | --- |
| *Predictors* | *Estimates* | *CI* | *p* | *Estimates* | *CI* | *p* | *Estimates* | *CI* | *p* |
| (Intercept) | 1.29 | 1.06 – 1.52 | **<0.001** | 1.29 | 1.06 – 1.52 | **<0.001** | 1.30 | 1.07 – 1.53 | **<0.001** |
| CTQ_PA | -0.04 | -0.06 – -0.03 | **<0.001** | -0.04 | -0.06 – -0.03 | **<0.001** | -0.03 | -0.05 – -0.01 | **0.001** |
| Country-level CTQ_PA | -0.00 | -0.11 – 0.10 | 0.932 | -0.00 | -0.11 – 0.10 | 0.933 | -0.01 | -0.12 – 0.09 | 0.790 |
| FCQ |  |  |  | 0.00 | -0.00 – 0.01 | 0.454 | 0.00 | -0.00 – 0.01 | 0.443 |
| Sex |  |  |  | 0.05 | -0.04 – 0.13 | 0.264 | 0.05 | -0.04 – 0.13 | 0.272 |
| PHQ-9 |  |  |  | -0.01 | -0.01 – 0.00 | 0.087 | -0.01 | -0.01 – 0.00 | 0.074 |
| MINI-SPIN |  |  |  | 0.02 | 0.01 – 0.04 | **<0.001** | 0.02 | 0.01 – 0.04 | **<0.001** |
| ITQ – PTSD |  |  |  | -0.00 | -0.01 – 0.00 | 0.347 | -0.00 | -0.01 – 0.00 | 0.350 |
| **Random Effects** | | | | | | | | | |
| σ^2^ | 0.99 | | | 0.99 | | | 0.98 | | |
| τ_00_ | 0.35 _Country_ | | | 0.35 _Country_ | | | 0.35 _Country_ | | |
| τ_11_ |  | | |  | | | 0.00 _Country.CTQ_PA_ | | |
| ρ_01_ |  | | |  | | | -0.73 _Country_ | | |
| ICC | 0.26 | | | 0.26 | | | 0.27 | | |
| Marginal R^2^ | 0.012 | | | 0.016 | | | 0.012 | | |

# Note. CTQ = Childhood Trauma Questionnaire, PA = Physical Abuse, PHQ = Patient Health Questionnaire, FCQ = Fear of the Coronavirus Questionnaire, PHQ-9 = Patient Health Questionnaire – 9, MINI-SPIN = Mini-Social Phobia Inventory, ITQ = International Trauma Questionnaire, ICC = Intraclass correlation

**Supplementary Table 5.** Model summary of linear mixed model analyses for Hypothesis # 4 – Sexual Abuse

|  | **Model 1**  **Random Intercept** | | | **Model 2**  **+ Covariates** | | | **Model 3**  **+ Random Slope CTQ** | | |
| --- | --- | --- | --- | --- | --- | --- | --- | --- | --- |
| *Predictors* | *Estimates* | *CI* | *p* | *Estimates* | *CI* | *p* | *Estimates* | *CI* | *p* |
| (Intercept) | 1.30 | 1.07 – 1.53 | **<0.001** | 1.30 | 1.07 – 1.53 | **<0.001** | 1.30 | 1.07 – 1.53 | **<0.001** |
| CTQ_SA | -0.01 | -0.02 – -0.00 | **0.003** | -0.01 | -0.02 – -0.00 | **0.019** | -0.01 | -0.02 – 0.00 | 0.072 |
| Country-level CTQ_SA | 0.03 | -0.09 – 0.14 | 0.646 | 0.03 | -0.09 – 0.14 | 0.645 | 0.03 | -0.09 – 0.14 | 0.648 |
| FCQ |  |  |  | 0.00 | -0.00 – 0.01 | 0.503 | 0.00 | -0.00 – 0.01 | 0.489 |
| Sex |  |  |  | 0.08 | -0.01 – 0.16 | 0.067 | 0.08 | -0.00 – 0.16 | 0.065 |
| PHQ-9 |  |  |  | -0.01 | -0.02 – -0.00 | **0.015** | -0.01 | -0.02 – -0.00 | **0.015** |
| MINI-SPIN |  |  |  | 0.02 | 0.01 – 0.04 | **<0.001** | 0.02 | 0.01 – 0.04 | **<0.001** |
| ITQ – PTSD |  |  |  | -0.01 | -0.01 – 0.00 | 0.108 | -0.01 | -0.01 – 0.00 | 0.099 |
| **Random Effects** | | | | | | | | | |
| σ^2^ | 1.00 | | | 1.00 | | | 1.00 | | |
| τ_00_ | 0.35 _Country_ | | | 0.35 _Country_ | | | 0.35 _Country_ | | |
| τ_11_ |  | | |  | | | 0.00 _Country.CTQ_SA_ | | |
| ρ_01_ |  | | |  | | | -0.21 _Country_ | | |
| ICC | 0.26 | | | 0.26 | | | 0.26 | | |
| Marginal R^2^ | 0.002 | | | 0.007 | | | 0.007 | | |

*Note.* CTQ = Childhood Trauma Questionnaire, SA = Sexual Abuse, PHQ = Patient Health Questionnaire, FCQ = Fear of the Coronavirus Questionnaire, PHQ-9 = Patient Health Questionnaire – 9, MINI-SPIN = Mini-Social Phobia Inventory, ITQ = International Trauma Questionnaire, ICC = Intraclass correlation.

**Supplementary Table 6.** Model summary of linear mixed model analyses for Hypothesis # 4 – Emotional Abuse

|  | **Model 1**  **Random Intercept** | | | **Model 2**  **+ Covariates** | | | **Model 3**  **+ Random Slope CTQ** | | |
| --- | --- | --- | --- | --- | --- | --- | --- | --- | --- |
| *Predictors* | *Estimates* | *CI* | *p* | *Estimates* | *CI* | *p* | *Estimates* | *CI* | *p* |
| (Intercept) | 1.27 | 1.04 – 1.49 | **<0.001** | 1.27 | 1.04 – 1.49 | **<0.001** | 1.27 | 1.04 – 1.50 | **<0.001** |
| CTQ_EA | -0.01 | -0.02 – -0.00 | **0.017** | -0.01 | -0.02 – 0.00 | 0.110 | -0.00 | -0.02 – 0.01 | 0.392 |
| Countrylevel_CTQ_EA | 0.06 | -0.02 – 0.14 | 0.141 | 0.06 | -0.02 – 0.14 | 0.141 | 0.05 | -0.03 – 0.13 | 0.207 |
| FCQ |  |  |  | 0.00 | -0.00 – 0.01 | 0.530 | 0.00 | -0.00 – 0.01 | 0.522 |
| Sex |  |  |  | 0.07 | -0.01 – 0.16 | 0.077 | 0.08 | -0.01 – 0.16 | 0.074 |
| PHQ-9 |  |  |  | -0.01 | -0.02 – -0.00 | **0.032** | -0.01 | -0.02 – -0.00 | **0.027** |
| MINI-SPIN |  |  |  | 0.02 | 0.01 – 0.04 | **<0.001** | 0.02 | 0.01 – 0.04 | **<0.001** |
| ITQ – PTSD |  |  |  | -0.01 | -0.01 – 0.00 | 0.083 | -0.01 | -0.01 – 0.00 | 0.080 |
| **Random Effects** | | | | | | | | | |
| σ^2^ | 1.00 | | | 1.00 | | | 1.00 | | |
| τ_00_ | 0.33 _Country_ | | | 0.33 _Country_ | | | 0.33 _Country_ | | |
| τ_11_ |  | | |  | | | 0.00 _Country.CTQ_EA_ | | |
| ρ_01_ |  | | |  | | | -0.84 _Country_ | | |
| ICC | 0.25 | | | 0.25 | | | 0.25 | | |
| Marginal R^2^ | 0.006 | | | 0.011 | | | 0.009 | | |

*Note.* CTQ = Childhood Trauma Questionnaire, EA = Emotional Abuse, PHQ = Patient Health Questionnaire, FCQ = Fear of the Coronavirus Questionnaire, PHQ-9 = Patient Health Questionnaire – 9, MINI-SPIN = Mini-Social Phobia Inventory, ITQ = International Trauma Questionnaire, ICC = Intraclass correlation.
